# Enhancement of Light Efficiency of Deep-Ultraviolet Light-Emitting Diodes by Encapsulation with a 3D Photonic Crystal Reflecting Layer

**DOI:** 10.3390/nano14110983

**Published:** 2024-06-05

**Authors:** Chun-Feng Lai, Chun-Peng Lin, Yu-Chun Lee

**Affiliations:** 1 Department of Photonics, Feng Chia University, Taichung 407, Taiwan; 2 Lextar Electronics Corp., Hsinchu Science Park, Hsinchu 30075, Taiwan; alu.lin@lextar.com (C.-P.L.); adam.lee@lextar.com (Y.-C.L.)

**Keywords:** UVC, LED, photonic crystals, reflector, photonic band gap

## Abstract

Recently, UVC LEDs, which emit deep ultraviolet light, have found extensive applications across various fields. This study demonstrates the design and implementation of thin films of three-dimensional photonic crystals (3D PhCs) as reflectors to enhance the light output power (LOP) of UVC LEDs. The 3D PhC reflectors were prepared using the self-assembly of silica nanospheres on a UVC LED lead frame substrate via the evaporation-induced method (side) and the gravitational sedimentation method (bottom), respectively. These PhCs with the (111) crystallographic plane were deposited on the side wall and bottom of the UVC LED lead frame, acting as functional materials to reflect UVC light. The LOP of UVC LEDs with 3D PhC reflectors at a driving current of 100 mA reached 19.6 mW. This represented a 30% enhancement compared to commercial UVC LEDs with Au-plated reflectors, due to the UVC light reflection by the photonic band gaps of 3D PhCs in the (111) crystallographic plane. Furthermore, after aging tests at 60 °C and 60% relative humidity for 1000 h, the relative LOP of UVC LEDs with 3D PhC reflectors decreased by 7%, which is better than that of commercial UVC LEDs. Thus, this study offers potential methods for enhancing the light output efficiency of commercial UVC light-emitting devices.

## 1. Introduction

At present, deep-ultraviolet light-emitting diodes (UVC LEDs), with emission wavelengths ranging from 200 nm to 280 nm, are of significant interest in solid-state lighting due to their small size, energy efficiency, long lifespan, stable performance, and environmental friendliness. Moreover, they find a variety of industrial applications, including water purification, environmental maintenance, medical treatment, food processing, livelihood improvement, the semiconductor equipment industry, and other fields [1,2,3,4]. Overall, the industrial applications of UVC LEDs contribute significantly to improving public health, ensuring product safety, and maintaining hygiene standards across various sectors. Unfortunately, UVC LEDs are limited by epitaxial and packaging technologies; consequently, commercial UVC LEDs mostly exhibit low external quantum efficiency and light output power (LOP), greatly limiting their applications [5]. Therefore, improving the light output efficiency (LOE) of UVC LEDs is crucial [6,7,8].

Another crucial aspect in improving the LOE of UVC LEDs is the reflective layer. Currently, commercial UVC LED lead frames use gold (Au) plating as a reflector; however, the reflectivity of Au drops dramatically to below 30% at wavelengths below 300 nm. Au is frequently employed as a packaging material for UVC LED lead frames owing to its stability, superb heat dissipation properties, and high thermal conductivity. Nevertheless, Au displays inadequate reflection of UVC light, consequently diminishing the LOP of UVC LEDs. Although alumina (Al) exhibits high reflectivity in the UVC region, it easily oxidizes, thereby reducing its reflectance. Therefore, materials with high reflectivity are still desired for UVC lighting devices. Recently, Shih et al. proposed the use of Al-Co-Zn-Ni high-entropy alloy films as UVC reflectors, which exhibit an average reflectance of 70% across the UVC wavelength range [9]. Wang et al. proposed enhancing the light extraction of UVC LEDs using an epoxy molding compound with a metallized reflector [10]. Therefore, enhancing the reflectance of the UVC LED lead frame will increase the LOP of UVC LEDs.

Three-dimensional photonic crystals (3D PhCs), created through artificial fabrication, consist of periodic crystal structures that can reflect light within specific photonic band gaps (PBGs) due to periodic fluctuations in the refractive index within their nanostructures [11,12,13,14,15,16,17,18,19,20]. This study develops a technique to enhance the LOP of UVC LEDs. The 3D PhCs, utilizing silica nanospheres deposited onto the UVC LED lead frame, increase the LOP due to their PBGs. As a reflective layer, the 3D PhCs can reflect UVC light and decrease UVC light absorption by the Au reflector.

## 2. Materials and Methods

### 2.1. Experimental Materials

Commercial UVC LEDs with a 4040 lead frame and Au plating were procured from Lextar Electronics Co. Ltd. (Hsinchu, Taiwan). Silica nanosphere powders were obtained from Shengsen Nano Tech. Co. Ltd. (Taichung, Taiwan). Absolute ethanol (EtOH, 99.99%) was used as a solvent to disperse silica nanospheres for the assembly of 3D PhC structures and was obtained from JT Baker Co. (New Jersey, NJ, USA).

### 2.2. UVC LED Lead Frame with 3D PhC Reflector

**Scheme 1:**
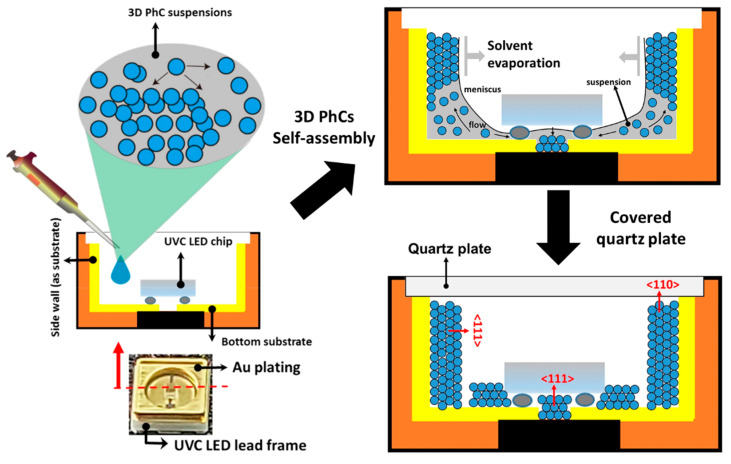
Illustration depicting the deposition of 3D PhCs onto the packaging of UVC LEDs.

Scheme 1 shows the 3D PhCs deposited onto the UVC LED lead frame. For this study, approximately 1.0 μL of 3D PhC suspension, with a concentration of 37 wt%, was applied onto the UVC LED lead frame using a micropipette to obtain the nearly close-packed face-centered cubic (*fcc*) crystal structure of 3D PhCs. The silica nanosphere suspensions were applied onto the UVC LED lead frame and subsequently placed in an oven set at a constant temperature of 50 °C for 2 h. Because the UVC LED lead frames have a deep round groove, the fabrication of 3D PhC reflective films was accomplished on the side wall substrate based on the evaporation-induced self-assembly method [21,22] and on the bottom substrate based on the gravitational sedimentation method [14,15]. This facilitated the assembly of silica nanospheres into a highly crystalline arrangement characterized by *fcc* crystals on UVC LED lead frames. Afterward, the oven temperature was raised to 80 °C for 30 min, effectively enhancing the rigidity of the 3D PhC reflective layer. Finally, the UVC LED lead frame was covered with a quartz flat packaging to provide physical and chemical protection. To mitigate the effects of moisture and environmental factors, the 3D PhCs were encapsulated with a quartz cover, effectively isolating the 3D PhCs from external humidity and improving the overall stability of the assembly. Additionally, the fabrication process involved securing the UVC LEDs onto the substrate first, ensuring their position and connection were stable. Afterward, the 3D PhCs were grown on the already fixed UVC LEDs, as illustrated in the process flow of Scheme 1. This method was chosen to prevent any adverse impact on the UVC LEDs’ position and performance during the 3D PhCs’ growth process.

### 2.3. Characterizations

The morphologies of the 3D PhCs were examined using a field-emission scanning electron microscope (FESEM; Hitachi, Tokyo, Japan). Reflectance measurements of the 3D PhCs were determined using an HR2000 spectrometer and calculated using SpectraSuite software (Ocean Insight, Orlando, FL, USA). A Y-type fiber-coupled 25 W deuterium lamp served as the UVC light source. The angular-resolved reflectance setup collected light using a spectrophotometer with a charge-coupled device (Jobin Yvon; Horiba, California, CA, USA). A fiber-coupled deuterium lamp was used as the UVC light source, and a double-motorized rotation stage collected light as a function of the zenith angle with a resolution of 1°. A UVC-enhanced mirror served as the standard for reflectance measurements. The characteristics of LOP versus current were measured using an irradiance integration sphere (FOIS-1) with a spectrometer (HR2000; Ocean Insight, Orlando, FL, USA). The aging tests were conducted within a test chamber (MHK–1680, Terchy Environmental Tech. Ltd., Nantou, Taiwan) set at 60 °C and 60% relative humidity (RH) for a duration of 1000 h. A vortex mixer with a horizontal oscillator was used to test for mechanical durability.

## 3. Results

### 3.1. The 3D PhC Nanostructures on the UVC LED Lead Frame

**Figure 1:**
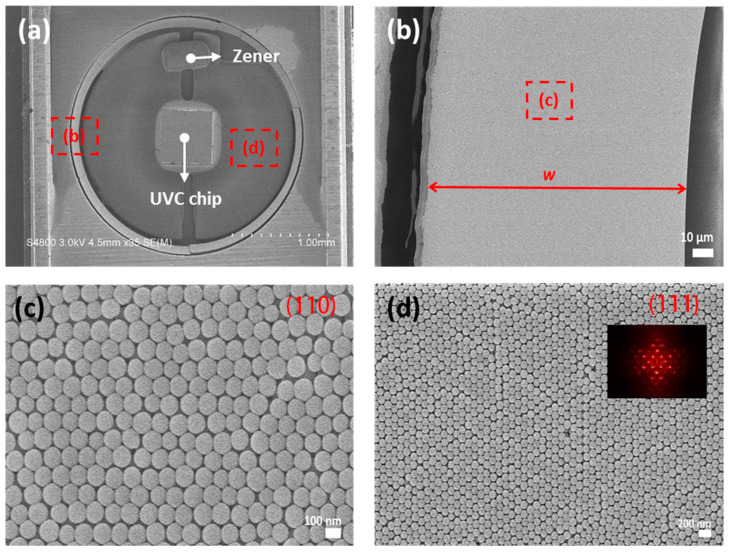
FESEM images of the 3D PhCs (**a**) deposited on the UVC LED lead frame, (**b**) forming a circular wall, (**c**) prepared using silica nanospheres with *D_avg_* of 124 nm on the side wall substrate (the inset shows the (110) lattice plane), and (**d**) revealing that the nanospheres are arranged in a well-organized (111) plane with *fcc* structures on the bottom substrate. The inset displays short-range ordered structures, as evidenced by fast Fourier transformation images.

FESEM was employed in this study to scrutinize the crystalline structure of the resulting 3D PhCs on the UVC LED lead frame. Silica nanospheres for the 3D PhCs were prepared to reflect UVC light, with mean diameters (*D*_avg_ ± standard deviation) of approximately 124 ± 10 nm and a polydispersity index (PDI) of about 0.08 (Figure S1). The 3D PhCs were deposited onto the UVC LED lead frame. Figure 1a presents the top view of the UVC LED packaged with the 3D PhC reflector films. Figure 1b shows the 3D PhCs deposited on the side wall substrate of the lead frame due to evaporation-induced self-assembly, forming a circular wall on the side wall substrate with a width (*w*) of approximately 110 μm and a height (*h*) of approximately 400 μm (Figure S2). The 3D PhCs featuring *fcc* crystal structures were cultivated through vertical sedimentation, with their top surfaces on the side wall and side substrate aligned parallel to the (110) and (111) crystallographic planes, respectively, as shown in Figure 1c and Figure S2c,d. Figure 1c depicts high-magnification FESEM images of the 3D PhCs produced using silica nanospheres. In addition, 3D PhCs with *fcc* structures were grown by gravitational sedimentation on the bottom substrate of UVC LED lead frames, with the surface parallel to the (111) crystallographic plane and a thickness (*t*) of approximately 15 μm (Figure 1d and Figure S2e). Figure 1d illustrates low-magnification FESEM images of the 3D PhCs. These images suggest that 3D PhCs can be manufactured within a relatively expansive domain featuring regular periodic structures. Furthermore, the diffraction pattern (inset Figure 1d) displayed a triangular lattice pattern, indicating that the 3D PhC reflection films were uniformly distributed with short-range ordered structures, aligning with the findings from FESEM observations.

### 3.2. The 3D PhC Reflective Layer Analyses

**Figure 2:**
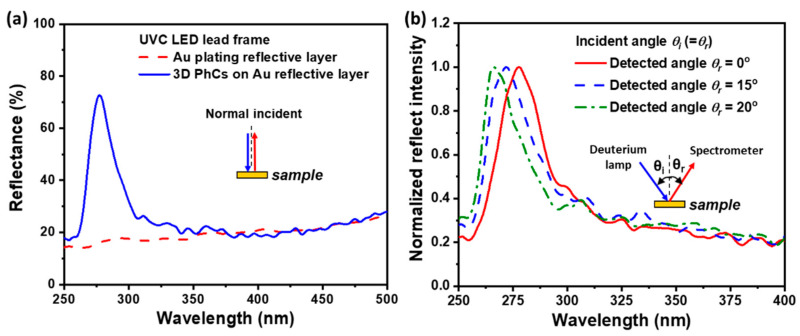
(**a**) The peak position of the reflectance spectra measured from the 3D PhCs is 277 nm. (**b**) Angle-resolved reflection measurement of 3D PhCs with normalized reflectivity.

Currently, bonding a UVC chip with an Au-plated lead frame is a standard commercial packaging method. However, the Au plating reflector often has surface impurities and areas of roughness. Consequently, the reflectance of the Au-plated lead frame in the UVC region is below 20%, as illustrated in Figure 2a. In contrast, the reflectance and the reflection wavelength (*λ*_R_) of 3D PhCs at a normal incidence ($\theta_{i}$ = $0^{\circ }$) are measured as 73% and 277 nm, respectively. The reflection spectrum has a full width at half maximum (FWHM) of approximately 21 nm. Additionally, 3D PhCs are known to possess PBG properties in the (111) crystallographic plane. Therefore, the PBG of 3D PhCs was measured using UVC reflection spectrometry and calculated based on the Bragg–Snell law.

The reflection wavelength (*λ*_R_) of the Bragg–Snell law was calculated using Equation (1) [19]:

(1) $$\lambda_{R}=2d_{111}\sqrt{{n_{eff}}^{2}-{sin}^{2}\theta_{i}}$$

where *λ*_R_ is 277 nm, *d*_111_ is the inter-planar spacing between the (111) crystallographic plane, and *n_eff_* is the effective refractive index of the crystalline lattice.

In the 3D PhC structure, the *n*_eff_ of the crystalline lattice satisfied Equation (2):

(2) $${n_{eff}}^{2}={n_{silica}}^{2}f_{silica}+{n_{air}}^{2}\left[1-f_{silica}\right]$$

where *n*_silica_ = 1.45 and *n*_air_ = 1.0 represent the refractive indices of the silica nanospheres and air, respectively. In this study, the sedimentation of 3D PhCs on a lead frame substrate resulted in a nearly close-packed face-centered cubic (*fcc*) crystal structure with a volume fraction of nanospheres (*f*_silica_) below 74 vol%. Therefore, the real value of *f*_silica_ can be calculated based on the Bragg–Snell equation using the measured *λ*_R_ [23]. According to ref. [23], the *n*_eff_ and *f*_silica_ are 1.31 and 66 vol%, respectively. In addition, we have included an analysis of the uncertainties associated with the calculation based on the reflection measurements. The primary sources of uncertainty in our calculation stem from the following factors: (a) *D*_avg_ was measured to be 124 nm with a standard deviation of approximately 10 nm. (b) The *n*_eff_ was calculated to be 1.31 with an estimated uncertainty of ±0.01. (c) The spectrometer and other optical measurement equipment introduce a systematic error, estimated to be ±1 nm. Therefore, the calculated *λ*_R_ is approximately 277 nm with an uncertainty of ±5 nm. This uncertainty analysis has been included to provide a comprehensive understanding of the precision of our measurements. This confirms the measured reflection wavelength peak results, as shown in Figure 2a, indicating that light waves cannot propagate within this region. In addition, we have included the calculated results (Figure S3) to assess the dependence of *λ*_R_ on the incident angle ($\theta_{i}$). The measured results are very consistent with the theoretical Bragg–Snell law calculation values.

To investigate the PBG of 3D PhCs, angular-resolved reflection was evaluated. The angle-resolved experimental setup is shown in the inset of Figure 2b. Collimated broadband UVC light from a deuterium lamp was incident on the sample at an angle $\theta_{i}$ from the surface normal. Reflected light was collected by a lens and focused onto an optical fiber connected to a spectrometer. The angle-resolved specular reflection was measured in the $\theta_{i}$ – $\theta_{r}$ geometry, as depicted in the experimental setup inset in Figure 2b. The reflection measurement results revealed that the PBG shifted towards shorter wavelengths as the detection angles ($\theta_{r}$) increased, indicating the impossibility of light wave propagation within this region. Additionally, the angular-resolved reflectance of 3D PhCs was measured using a UVC-enhanced mirror as the standard. The reflectance of 3D PhCs decreased from 73% ($\theta_{i}=0^{\circ }$) to 27% ($\theta_{i}=25^{\circ }$) as the incident angle increased, as shown in Figure S4.

### 3.3. Optical Properties of UVC LEDs with 3D PhC Reflective Layer

**Figure 3:**
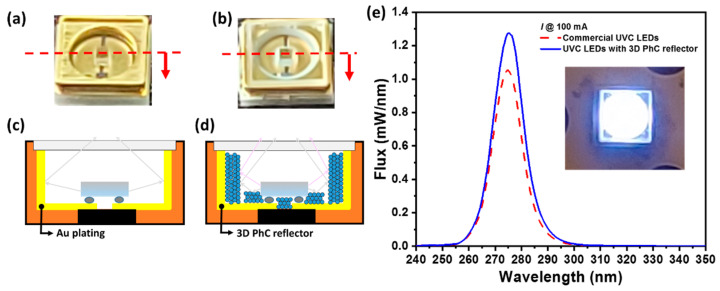
Optical and schematic cross-section images of (**a**,**c**) commercial UVC LEDs and (**b**,**d**) UVC LEDs with 3D PhC reflectors, respectively. (**e**) The luminescence spectra of all samples are shown under a driving current of 100 mA. The inset shows the luminescence of a UVC LED with a 3D PhC reflector.

In this section, electroluminescence (EL) measurements were conducted by injecting a continuous current into all LEDs at room temperature. The electrical and optical properties, as well as the output power, were determined using a calibrated integrating sphere. The peak wavelength of commercial UVC LEDs and UVC LEDs with 3D PhCs was found to be the same, 275 nm, with an FWHM of 13 nm and 14 nm at a driving current of 100 mA, respectively (Figure 3). Upon deposition of the 3D PhC reflective film, the LOP of UVC LEDs was enhanced. This outcome indicates that the 3D PhC reflector improves the LOE by reflecting small-angle light ($0^{\circ }$~$20^{\circ }$) with PBG properties, as depicted in Figure 2b. Furthermore, Figure 3 illustrates the high reflectance layer obtained by depositing 3D PhCs on a UVC LED lead frame instead of the traditional Au plating. The high reflectance of 3D PhCs is markedly enhanced compared to the commercial Au plating reflector, indicating significant potential for application in UVC LEDs. Based on the spectrometer measurement equipment having a ±1 nm systematic error, in this study, the 3D PhCs exhibited no effect on the FWHM of the luminescence spectrum of UVC LEDs. Due to the UVC LEDs having a 14 nm FWHM (emission peak at 275 nm), the 3D PhCs can reflect the UVC light only at incident angles from $0^{\circ }$ (*λ*_R_ = 277 nm, R% = 73%) to $20^{\circ }$ (*λ*_R_ = 266 nm, R% = 27%) according to Figure 2b. Therefore, we designed the PBG of 3D PhCs for the 275 nm emission peak of UVC LEDs, with the maximum reflectance occurring at normal incidence.

**Figure 4:**
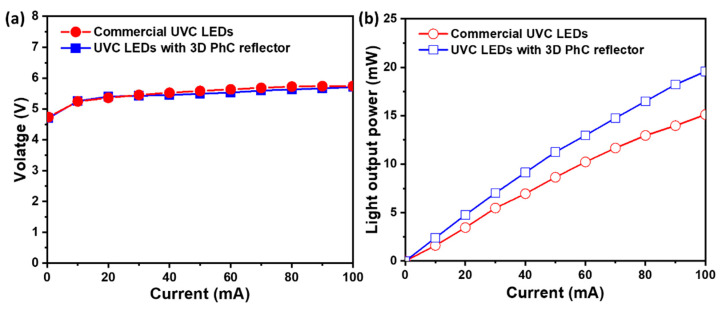
(**a**) Displays the voltage–current characteristics of the UVC LED with the 3D PhC reflector compared to commercial UVC LEDs. (**b**) Illustrates the LOP of UVC LEDs packaged with the 3D PhC reflector and the commercial Au plating package.

The integration sphere was used to measure the optical properties of two light sources: UVC LEDs with 3D PhCs and commercial UVC LEDs. In Figure 4a, it is observed that all devices reached their maximum rated current at approximately 4.7 V, consistent with the anticipated AlGaN composition. The LOP of UVC LEDs, manufactured with various packaging materials, was measured across forward currents ranging from 0 mA to 100 mA, as illustrated in Figure 4b. Clearly, the LOP increases with increasing current. Importantly, the LOP of UVC LEDs packaged with the 3D PhC reflector surpasses that of UVC LEDs packaged with Au plating. Specifically, the LOP of UVC LEDs was improved by 30% (from 15.1 mW to 19.6 mW) with the implementation of 3D PhC reflective films under 100 mA. This increase in LOP is attributed to the light reflection from the PBG of the 3D PhC reflector.

**Figure 5:**
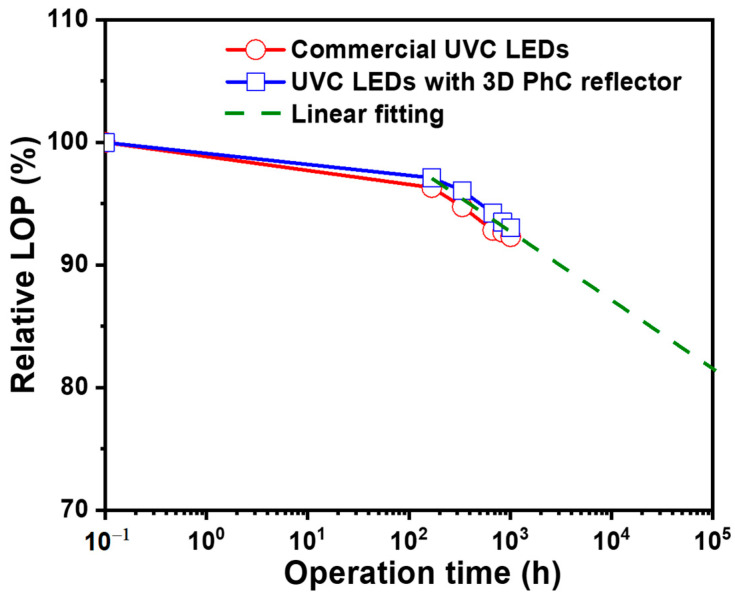
Relative LOP of UVC LEDs during the aging tests conducted at 60 °C and 60% RH for 1000 h.

Aging tests were conducted to evaluate the reliability of the 3D PhC reflector package, which is crucial for UVC LED device applications. In lighting contexts, reliability analysis testing often determines the operational time until the LOP of UVC LED devices decreases to 70% of the initial value (L70). We conducted a wet, high-temperature operating life test to evaluate the reliability of the UVC LEDs, maintaining a temperature of 60 °C and relative humidity (RH) of 60%, with a forward current of 100 mA, over 1000 h, comparing UVC LEDs containing 3D PhC samples to commercial UVC LEDs with Au plating. Figure 5 illustrates the normalized LOP as a function of time, where the LOP was normalized to the intensity after ten minutes of operation. During the 1000 h aging test, the LOP of UVC LEDs packaged with the 3D PhC reflector and Au plating decreased by 7% and 8%, respectively, as shown in Figure 5. Various optical power degradation mechanisms have been observed in UVC LEDs at different stages of degradation, including hillock-related and stress-induced defects [24,25]. The results of the 1000 h aging test indicated that UVC LEDs containing 3D PhC samples were nearly consistent with commercial UVC LEDs, and the 3D PhCs deposited on the UVC LED lead frame remained undamaged, demonstrating excellent reliability. Hence, we concluded that the decrease in the LOP of UVC LEDs with 3D PhCs is attributed to the LOP decline in the commercial UVC LED chip resulting from prolonged operation. Extrapolating the data from the last 1000 h, both devices have a projected L70 lifetime exceeding 10,000 h, meeting the industry’s minimum reliability standards [5].

Three-dimensional PhCs are known for their limited durability and stability. In this study, the 3D PhCs were stacked on a lead frame to serve as a UVC reflector, and their adhesion was tested for mechanical durability using the horizontal oscillator method. UVC LEDs with a 3D PhC reflecting layer underwent 24 h of horizontal oscillation, during which no significant shedding of 3D PhCs from the UVC LED lead frame substrate was observed (Figure S5). Even so, we aim to provide an optimal approach that may be implemented in the future. In previous research, we have demonstrated that infiltrating silicone materials into colloidal crystal channels can enhance the mechanical strength and stability of 3D PhCs [14]. Unfortunately, there is currently no optical adhesive that can withstand UVC light irradiation, which is necessary for UVC LEDs to pass the reliability test. Therefore, we can only temporarily encapsulate UVC LEDs with quartz plates to protect them from environmental degradation. In this research, we have demonstrated that using a 3D PhC reflective layer on the lead frame side substrate and bottom substrate of UVC LEDs can improve the LOP and reduce Au plating absorption. In the future, with the development of an anti-UVC optical adhesive, this technology could potentially be used in the commercialization of UVC LEDs.

## 4. Conclusions

In conclusion, this study successfully demonstrated the fabrication of UVC LEDs incorporating a 3D PhC reflecting layer with a high reflectance of 70%, resulting in a notable enhancement in LOP. Specifically, the LOP of the UVC LEDs with 3D PhCs reached 19.6 mW at 100 mA. Experimental findings revealed a 30% enhancement in LOP compared to the Au plating package at the same current. Additionally, during the aging tests conducted at 60 °C and 60% RH for 1000 h, the relative LOP of the UVC LEDs with 3D PhC reflector decreased by 7%. The 3D PhC reflector package offers a promising, cost-effective, and high-efficiency method for UVC devices. Overall, this study provides a promising technique for enhancing the LOP of commercial UVC LEDs.

## Data Availability

Data are contained within the article and Supplementary Materials.

## Supplementary Materials

The following supporting information can be downloaded at: https://www.mdpi.com/article/10.3390/nano14110983/s1, Figure S1: Particle size distribution calculated from FESEM pictures. (a) FESEM image and (b) histograms of particle size distribution of SNPs. Figure S2: (a) Optical image corresponding to (b) FESEM image of the circle wall and bottom sedimentation, showing (c) width (w), (d) height (h), and (e) thickness (t) of the 3D PhCs. Figure S3: The dependence of reflection wavelength on the incident angle for both measured results and theoretical calculation. Figure S4: Angle-resolved reflectance measurement of 3D PhCs. Figure S5. Optical images of UVC LEDs with 3D PhC reflectors before and after the mechanical durability test using a horizontal oscillator.
