# Supplementary material for: Enhancement of Light Efficiency of Deep-Ultraviolet Light-Emitting Diodes by Encapsulation with a 3D Photonic Crystal Reflecting Layer
Source: Nanomaterials (Basel). 2024 Jun 5;14(11):983. doi: 10.3390/nano14110983 (PMC11173468; doi:10.3390/nano14110983)
Supplement: Supplementary file 1 [file nanomaterials-14-00983-s001.zip › nanomaterials-2996264-supplementary.pdf]

Supporting Information

Enhancement of Light Efficiency of Deep-Ultraviolet Light-Emitting Diodes by Encapsulation with a 3D Photonic Crystal Reflecting Layer

Figure S1. Particle size distribution calculated from FESEM pictures. (a) FESEM image and (b) histograms of particle size distribution of silica nanospheres.

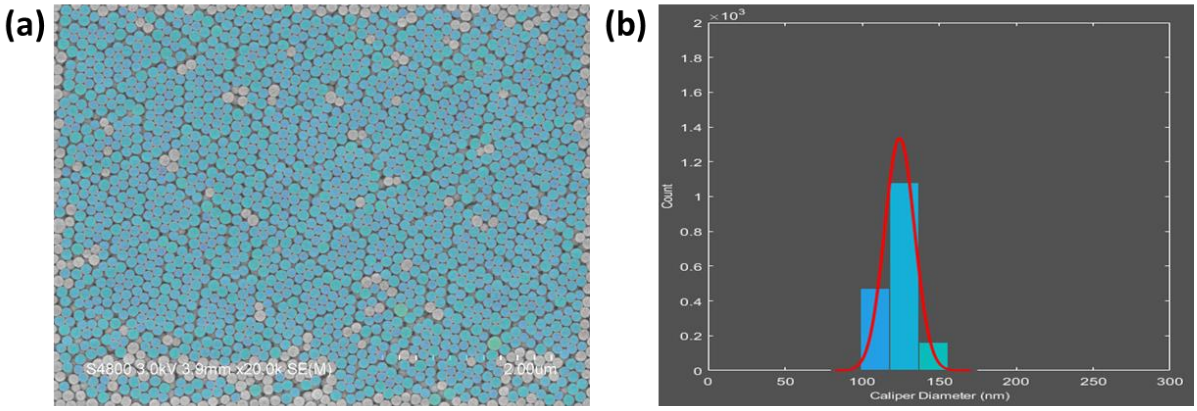

Figure S2. (a) Optical image corresponding to (b) FESEM image of the side wall substrate and bottom substrate sedimentation, showing (c) width (*w*), (d) height (*h*), and (e) thickness (*t*) of the 3D PhCs. Inset shows the crystallographic plane.

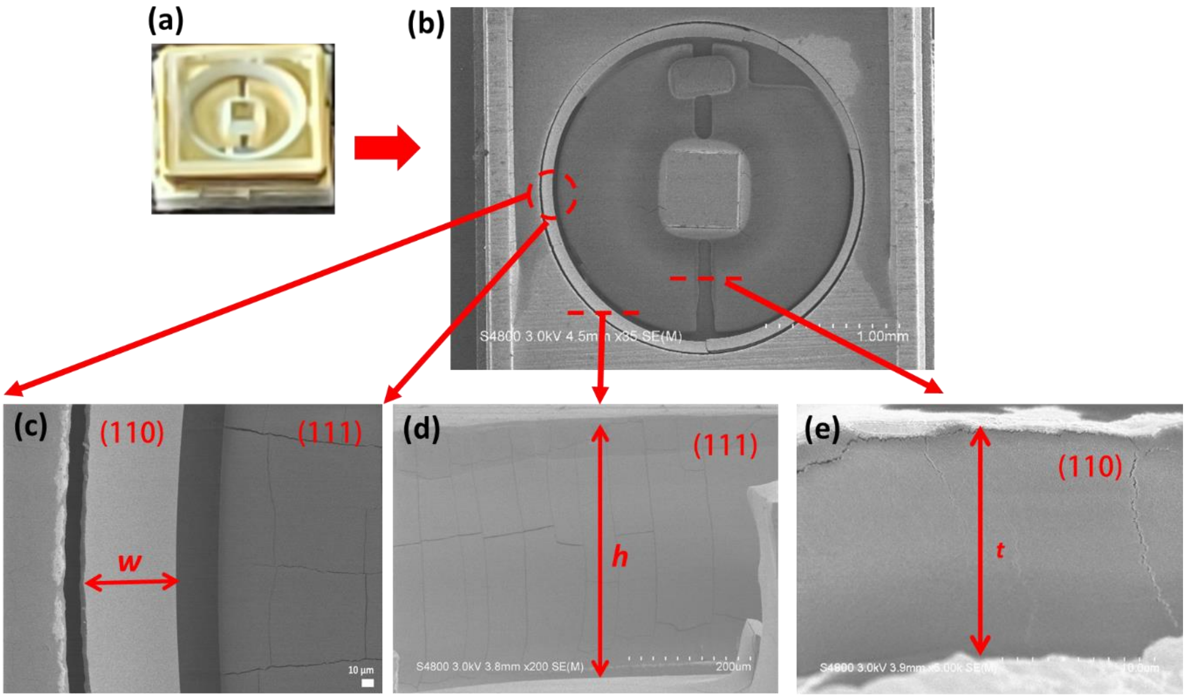

Figure S3. The dependence of reflection wavelength on the incident angle for both measured results and theoretical calculation.

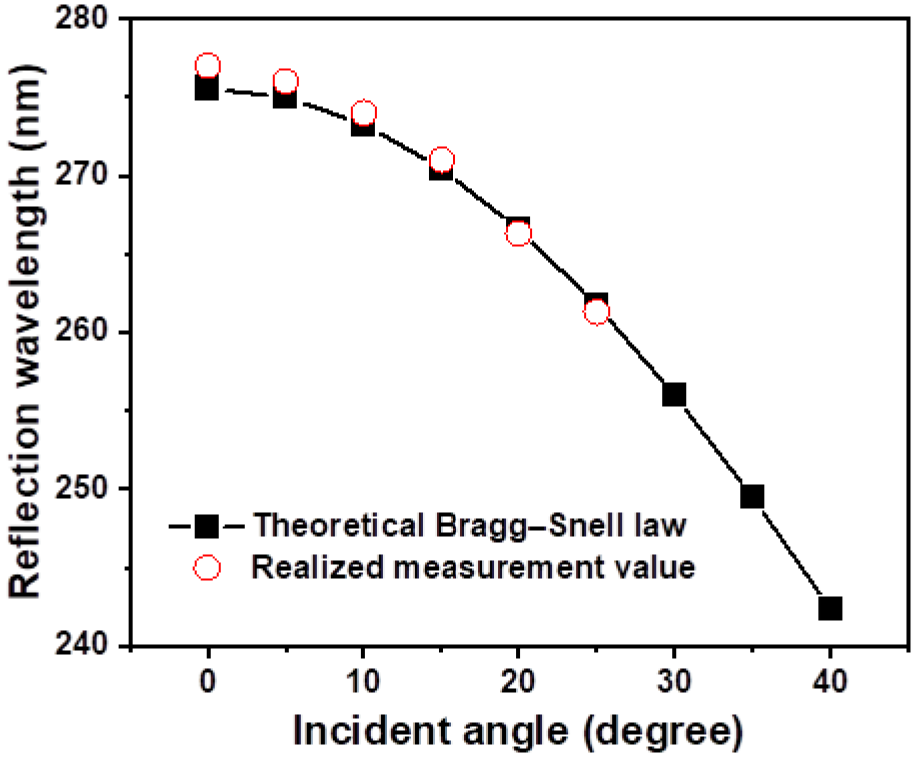

Figure S4. Angle-resolved reflectance measurement of 3D PhCs.

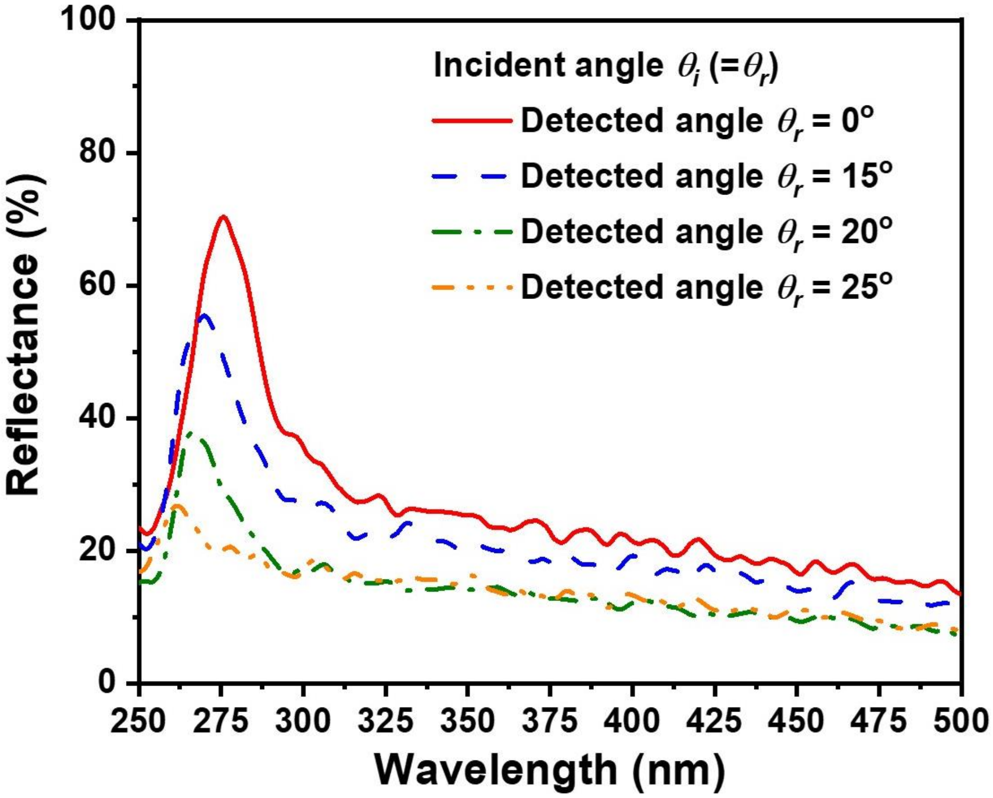

Figure S5. Optical images of UVC LEDs with 3D PhC reflectors undergoing a mechanical durability test from 0 hours to 24 hours using a horizontal oscillator.

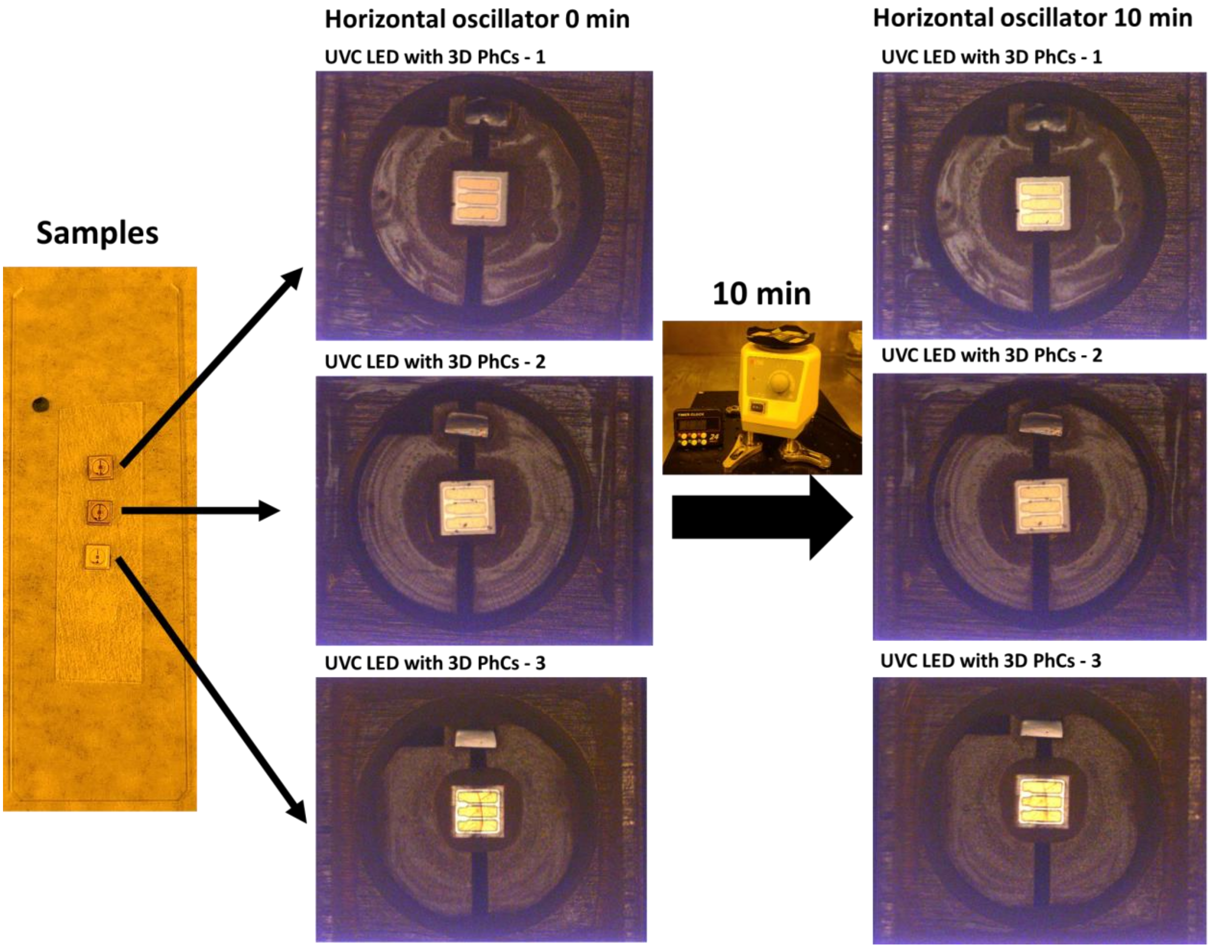

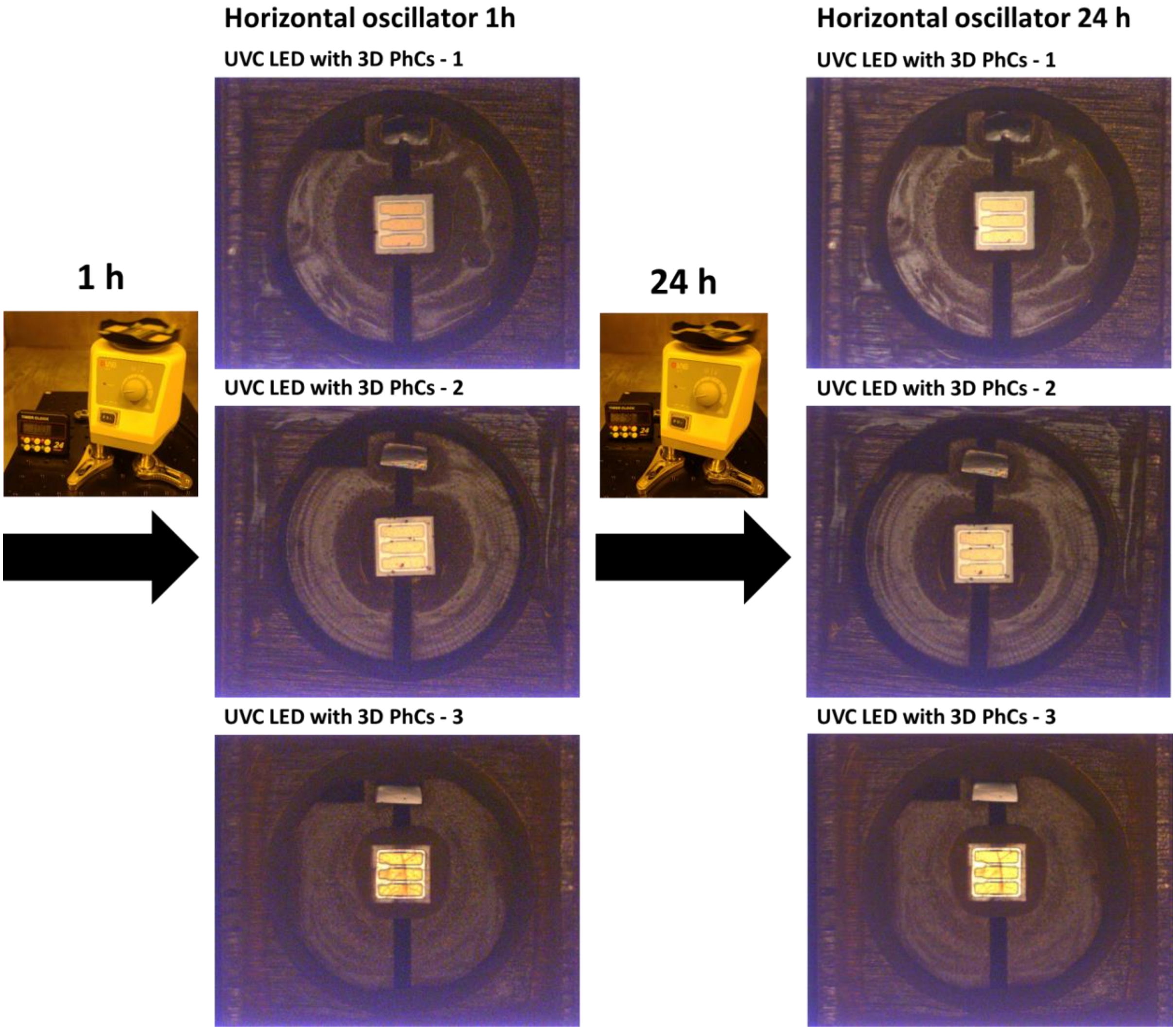
Horizontal oscillator 1h
UVC LED with 3D PhCs - 1
1 h
UVC LED with 3D PhCs - 2
UVC LED with 3D PhCs - 3
Horizontal oscillator 24 h
UVC LED with 3D PhCs - 1
24 h
UVC LED with 3D PhCs - 2
UVC LED with 3D PhCs - 3
